# Dr. Andre L. Cowles

**Published:** 1904-04

**Authors:** 


Dr. Andre L. Cowles, of Spartansburg, Pa., University of Buffalo, 1892, died February 18, 1904, aged 60 years.

